# Metastatic Colorectal Cancer as a Chronic Disease: Twelve-Year Survival After Initially Unresectable Bilobar Liver Metastases

**DOI:** 10.7759/cureus.105940

**Published:** 2026-03-26

**Authors:** Thomas Mathew, Steven Smith, Mathew George

**Affiliations:** 1 Joint Medical Programme, University of Newcastle, Newcastle, AUS; 2 Medical Oncology, Calvary Mater Newcastle Hospital, Newcastle, AUS

**Keywords:** chronic cancer management, colorectal liver metastases, conversion chemotherapy, long-term survival, metastasectomy, metastatic colorectal cancer

## Abstract

Metastatic colorectal cancer is usually associated with limited long-term survival, especially when the disease is extensive or initially considered unresectable. In such situations, survival extending beyond a decade is uncommon and typically confined to very carefully selected patients.

We report the case of a 61-year-old woman with sigmoid colon adenocarcinoma who developed extensive bilobar liver metastases that were initially not amenable to surgery. Over a disease course lasting 12 years, she experienced multiple recurrences involving the liver, lungs, and brain. Management included multi-line systemic chemotherapy, delayed hepatic resections after tumor downstaging, repeated locoregional ablative procedures, and participation in an early-phase immunotherapy trial. Despite substantial treatment-related toxicity, disease control was maintained for many years before eventual progression.

This case illustrates that metastatic colorectal cancer can occasionally follow a prolonged, relapsing course resembling a chronic illness. It emphasizes the potential value of repeated reassessment of resectability, integration of local therapies, and coordinated multidisciplinary care in selected patients.

## Introduction

Colorectal cancer is a leading cause of cancer-related morbidity and mortality worldwide, and a substantial proportion of patients either present with or later develop metastatic disease [1,2]. Although advances in systemic chemotherapy, biologic agents, and liver-directed therapies have improved outcomes, metastatic colorectal cancer remains incurable for most patients [1,2].

Long-term survival is most often observed in patients with a limited metastatic burden who can undergo complete resection of liver and/or lung metastases. In contrast, patients with extensive bilobar colorectal liver metastases that are initially considered unresectable usually have a poor prognosis, and survival beyond 10 years is rare [1-3]. Conversion chemotherapy may downstage disease and permit delayed resection; however, durable remission is achieved in only a minority of cases. Globally, colorectal cancer accounts for approximately 1.93 million new cases and 940,000 deaths annually, and approximately one-third of patients will develop metastatic disease at some point during their illness [4,5].

We report an unusual case of a 61-year-old woman with metastatic colorectal cancer with initially unresectable bilobar liver metastases - confirmed at operative exploration - in which survival exceeding 12 years was achieved through sustained chemosensitivity, repeated reassessment of resectability, aggressive local therapy, and coordinated multidisciplinary management.

This case is reported for several reasons. Twelve-year survival in the context of bilobar hepatic metastases confirmed unresectable at operative exploration is exceptionally rare and incompletely characterised in the literature. The patient had microsatellite-stable disease, deriving no benefit from immunotherapy, and the prolonged survival was achieved exclusively through chemotherapy, sequential surgical resection, and locoregional ablative strategies. Documentation of this case contributes to the evidence base supporting aggressive, longitudinal multimodal management in patients with metastatic colorectal cancer, and illustrates that metastatic disease of this extent and biology can, in exceptional circumstances, follow a chronic disease trajectory.

## Case presentation

**Figure 1:**
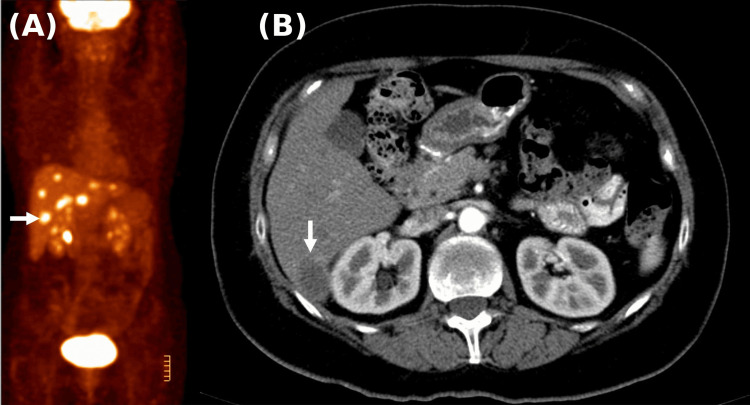
FDG-PET/CT imaging from 2010 demonstrating extensive bilobar hepatic metastases (A) Coronal FDG-PET image demonstrating multiple hypermetabolic foci throughout the liver (arrow), consistent with extensive bilobar metastatic disease. (B) Axial CT image showing a corresponding hypodense hepatic lesion in the right lobe of the liver (arrow), confirming metastatic involvement. FDG-PET/CT: fluorodeoxyglucose-positron emission tomography/computed tomography

A 61-year-old woman in her early 60s was diagnosed in June 2010 with sigmoid colon adenocarcinoma and underwent a high anterior resection. Pre-operative CT imaging demonstrated a cystic lesion in the right hepatic lobe only, with no evidence of confirmed metastatic disease at the time of surgery, and the pre-operative carcinoembryonic antigen (CEA) was 3.7 mcg/L. Histopathology demonstrated a moderately differentiated invasive adenocarcinoma measuring 35 × 25 mm, with serosal involvement (pT4a) and no lymph node involvement (0/13). There was no lymphovascular or perineural invasion, and molecular analysis confirmed KRAS/NRAS wild-type disease. On routine post-operative follow-up in November 2010, the CEA had risen to 46 mcg/L, and repeat CT of the abdomen demonstrated low-attenuating lesions in both hepatic lobes, with the largest measuring 2.8 cm in segment 6, confirming metastatic disease. The patient was entirely asymptomatic at this time; there were no symptoms attributable to hepatic involvement, and the metastases were detected solely on surveillance imaging and biochemical monitoring. The treating surgeon referred the patient to medical oncology, and she was first reviewed by a medical oncologist on 15 December 2010, at which time metastatic disease was identified and considered initially inoperable. The hepatobiliary surgeon subsequently performed an exploratory laparotomy, at which intraoperative assessment confirmed at least eight metastatic lesions distributed across seven of eight hepatic segments; liver resection was abandoned given the extent of bilobar disease. A fluorodeoxyglucose-positron emission tomography (FDG-PET) scan performed in 2010 demonstrated extensive bilobar hepatic metastases with multiple metabolically active lesions (Figure 1), confirming advanced metastatic disease (Table 1).

**Table 1:** Long-term disease control using sequential standard and trial-based therapies in metastatic MSS colorectal cancer (2010-2022) Timeline of treatments and clinical outcomes in a patient with metastatic microsatellite-stable (MSS) colorectal cancer over a 12-year period. FOLFOX: folinic acid/5-fluorouracil/oxaliplatin; FOLFIRI: folinic acid/5-fluorouracil/irinotecan; XELOX: capecitabine/oxaliplatin; XELIRI: capecitabine/irinotecan; FDG-PET: fluorodeoxyglucose positron emission tomography; CEA: carcinoembryonic antigen; mCRC: metastatic colorectal cancer; PFO: patent foramen ovale

Time Period	Treatment/Intervention	Clinical Events	Outcome
June 2010	High anterior resection for sigmoid colon adenocarcinoma (regional centre)	Pre-operative CT (June 2010): cystic lesion right hepatic lobe only - no confirmed metastases at surgery. Moderately differentiated adenocarcinoma, 35 x 25 mm, pT4a, N0 (0/13), KRAS/NRAS wild-type; no lymphovascular or perineural invasion. Pre-operative CEA 3.7 mcg/L.	Complete resection, clear margins. Patient under routine surgical follow-up.
November 2010	Routine post-operative surgical follow-up	CEA risen to 46 mcg/L. CT abdomen: low-attenuating lesions both hepatic lobes, largest segment 6 (2.8 cm). Metastatic disease confirmed on imaging.	Surgeon referred patient to medical oncology
December 2010	First medical oncology review (the treating medical oncologist, 15 December 2010)	Rising CEA and CT-confirmed hepatic metastases documented. Plan: PET scan and liver surgeon's opinion. Patient had appointment arranged at a tertiary hepatobiliary center.	Appropriate workup initiated promptly. No avoidable delay.
December 2010-January 2011	Hepatobiliary surgical assessment - exploratory laparotomy, a tertiary hepatobiliary center	Intraoperative assessment: at least eight metastatic lesions, seven of eight hepatic segments involved. Liver resection abandoned.	Open-and-close approach. Referred back to medical oncology for palliative systemic therapy.
February 2011	Portacath insertion; FOLFOX 6 plus bevacizumab commenced	Oncology letter 11 February 2011 confirmed liver lesions inoperable following abandoned surgery. Palliative chemotherapy FOLFOX 6 plus bevacizumab planned. Portacath arranged prior to the first cycle.	Chemotherapy commenced in February 2011.
February 2011-January 2012	FOLFOX 6 plus bevacizumab (oxaliplatin discontinued after planned treatment period)	Treatment well-tolerated. Oxaliplatin was discontinued after the planned cumulative exposure; no clinically significant peripheral neuropathy developed - a noteworthy feature of the case.	Radiologic resolution of hepatic metastases on January 2012 imaging.
January-May 2012	Treatment break	Clinical and radiologic surveillance.	Maintained disease control.
May 2012	First liver resection	Despite near-complete radiologic response, histopathology revealed a small residual focus of viable adenocarcinoma with extensive chemotherapy effect, confirming residual microscopic disease.	Clear surgical margins achieved.
July-October 2012	Capecitabine plus bevacizumab	Adjuvant therapy post-hepatic resection.	Disease progression identified.
November 2012-May 2013	Irinotecan plus cetuximab	Sufficient disease control achieved.	Enabled second liver resection.
May 2013	Second liver resection	Cetuximab discontinued shortly after due to an infusion reaction.	Successful surgical resection.
2013-2014	FOLFOX rechallenge, then capecitabine plus bevacizumab	Transition to maintenance therapy.	Stable disease on maintenance.
2014-2016	Maintenance capecitabine plus bevacizumab	Colonic fistula (resolved conservatively); embolic stroke (no permanent neurological deficit, exact aetiology undetermined - PFO considered as one possible contributing factor); pulmonary embolism.	Continued disease control despite comorbid events.
December 2016	Radiofrequency ablation (RFA)	RFA of oligometastatic lesions in the liver and lung.	Local disease control achieved.
January-September 2017	XELIRI plus bevacizumab	FDG-PET September 2017: recurrent metabolically active lesions in the liver and lungs.	Disease progression.
September 2017-April 2018	XELOX alternating with XELIRI based on toxicity and rising CEA	Further surgical intervention no longer feasible by April 2018.	Transient disease control.
April-December 2018	Cetuximab rechallenge, then FOLFIRI plus cetuximab	Cumulative chemotherapy toxicity.	Transient disease control.
January-June 2019	FOLFOX plus bevacizumab with stereotactic body radiotherapy to liver lesions	Local ablative therapy combined with systemic treatment.	Disease progression later in 2019.
Late 2019	Single-agent raltitrexed	Exhaustion of standard systemic therapy options.	Limited disease control.
December 2019-November 2020	Investigational trial: PG545 (pixatimod) plus nivolumab (immunotherapy for MSS mCRC)	April 2020: approaching partial response (38.8% reduction in liver lesions). August 2020: immune-mediated pneumonitis requiring hospitalisation and high-dose corticosteroids. October 2020: recurrent pneumonitis on rechallenge.	Partial radiologic response achieved; trial permanently discontinued due to drug-related pneumonitis.
Early 2021	Surveillance imaging	Disease progression with hepatic and osseous metastases, including skull involvement.	Progression post-trial discontinuation.
August 2021-January 2022	Second investigational trial: sorafenib plus MG010 (for MYC-amplified MSS CRC)	Day 168 (January 2022): overall stable disease; equivocal new pulmonary nodules; rising CEA.	Trial discontinued due to disease progression.
February 2022	Palliative cranial radiotherapy	Symptomatic brain metastasis with meningeal involvement. FDG-PET: extensive disease in the liver, lungs, and brain.	Palliative treatment for CNS disease.
May 2022	Supportive care	Further progression of intracranial, pulmonary, and hepatic disease with vasogenic oedema.	Corticosteroids and antiepileptic therapy initiated.
Mid-2022	FOLFIRI rechallenge	Given prior treatment-free interval and prolonged disease course.	Continued disease progression.
October 2022	End-of-life care	Death from complications of metastatic colorectal cancer.	12-year survival from initial diagnosis.

In February 2011, the medical oncologist confirmed the liver lesions were inoperable following the abandoned surgery and documented a plan to commence palliative chemotherapy with FOLFOX plus bevacizumab following Port-a-Cath insertion. Between February 2011 and January 2012, she received first-line systemic therapy with FOLFOX plus bevacizumab. Oxaliplatin was discontinued after the planned cumulative exposure; no clinically significant peripheral neuropathy developed.

Subsequent imaging in January 2012 demonstrated radiologic resolution of hepatic metastases, allowing a treatment break. In May 2012, she underwent her first liver resection, with histopathology revealing only a small residual focus of adenocarcinoma showing chemotherapy effect and clear margins. She received capecitabine plus bevacizumab from July to October 2012, before disease progression prompted initiation of irinotecan plus cetuximab in November 2012. This treatment resulted in sufficient disease control to permit a second liver resection in May 2013.

Cetuximab was discontinued shortly thereafter due to an infusion reaction, and she was treated with a rechallenge of folinic acid, fluorouracil, and oxaliplatin (FOLFOX), later transitioned to capecitabine plus bevacizumab. From 2014 to 2016, she remained on maintenance systemic therapy. During this period, she developed a colonic fistula, which resolved with conservative management without requiring surgical intervention or diversion; hepatic and renal function remained within acceptable parameters throughout. Significant comorbid events during this time included an embolic stroke, which resolved without permanent neurological deficit, and a pulmonary embolism; both were managed without long-term interruption of oncologic treatment.

She underwent radiofrequency ablation (RFA) of limited residual metastatic deposits in the liver and lung in December 2016, at a time when the disease burden was considered amenable to locoregional ablative therapy. In January 2017, systemic therapy was escalated to capecitabine and irinotecan (XELIRI) plus bevacizumab.

Disease progression was identified on FDG-PET imaging in September 2017, demonstrating recurrent metabolically active lesions in the liver and lungs. Treatment was subsequently modified between XELOX and XELIRI based on cumulative toxicity and rising CEA levels. By April 2018, further surgical intervention was no longer considered feasible. She subsequently underwent a cetuximab rechallenge, followed by FOLFIRI plus cetuximab, achieving transient disease control.

Between January and June 2019, she was treated with FOLFOX plus bevacizumab combined with stereotactic radiotherapy to liver lesions. Disease progression later that year led to treatment with single-agent raltitrexed. Given exhaustion of standard systemic options, she commenced a combination investigational immunotherapy trial with PG545 plus nivolumab in December 2019 for microsatellite-stable metastatic colorectal cancer.

After 16 weeks of investigational therapy, imaging in April 2020 demonstrated continued reduction in hepatic and pulmonary metastases, described as approaching a partial response, accompanied by a marked biochemical response. A confirmatory CT scan in May 2020 demonstrated a 38.8% reduction in target liver lesions, consistent with partial radiologic response. Treatment was initially well tolerated; however, in August 2020, she developed immune-mediated pneumonitis, requiring hospital admission and treatment with high-dose corticosteroids and antibiotics. Following clinical improvement, a treatment rechallenge was attempted in October 2020, but recurrent pneumonitis occurred, leading to permanent discontinuation of the trial in November 2020. Subsequent evaluation supported a drug-related pneumonitis, and she was managed with a prolonged corticosteroid taper.

Imaging in early 2021 demonstrated disease progression, with hepatic and osseous metastases, including involvement of the skull. In August 2021, she commenced a second investigational targeted therapy trial (sorafenib plus MG010) for MYC-amplified microsatellite-stable colorectal cancer. She remained on trial for several months. At day 168 of treatment in January 2022, imaging demonstrated overall stable disease compared with prior assessments, despite equivocal new pulmonary nodules and a rising CEA level. The trial was discontinued later that month due to disease progression.

**Figure 2:**
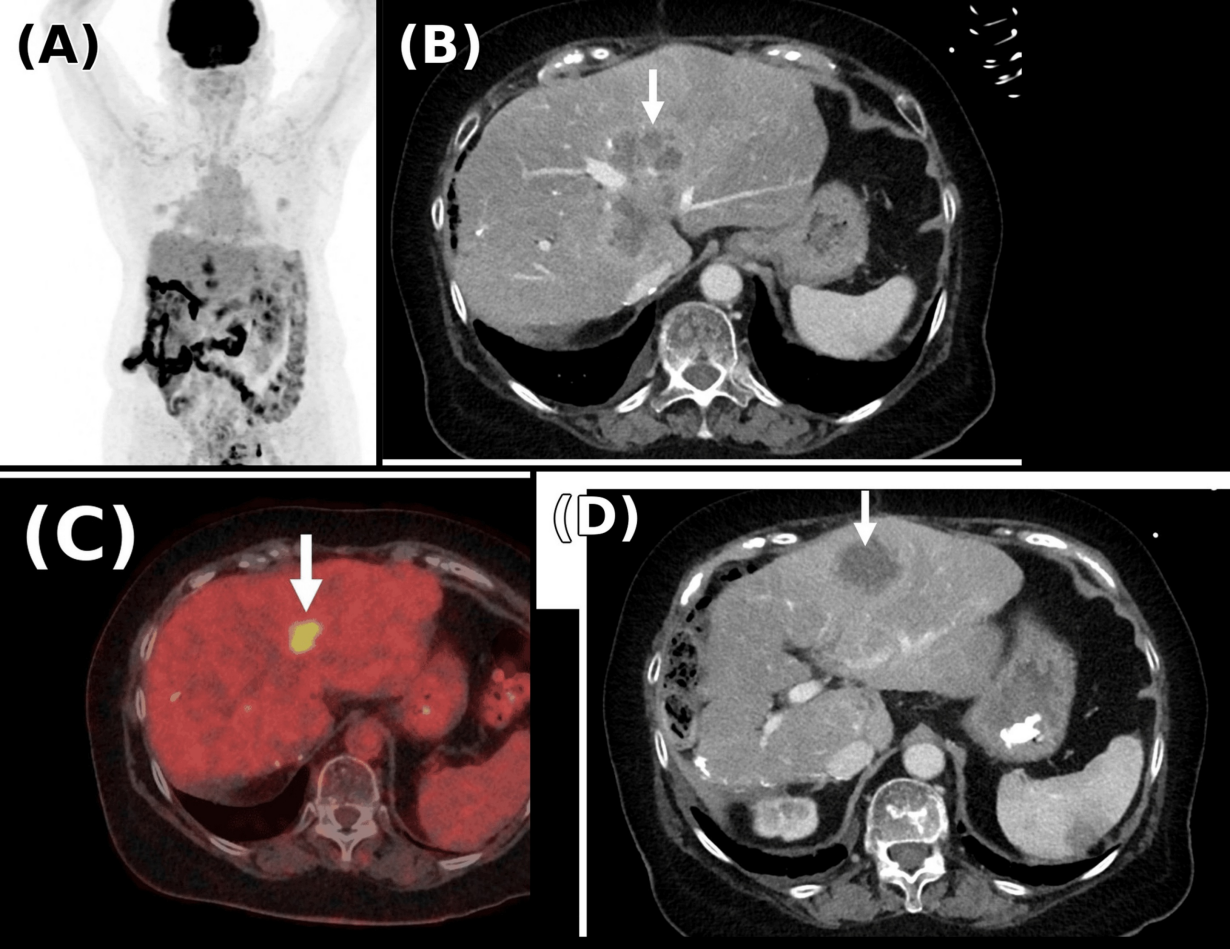
FDG-PET/CT imaging from February 2022 (A) Coronal whole-body FDG-PET showing widespread metabolic activity. (B) Axial CT showing hepatic metastatic deposits with post-ablation changes (arrow). (C) Axial FDG-PET/CT fusion showing a focal hypermetabolic hepatic lesion (arrow). (D) Axial CT at a lower level showing additional hepatic metastatic deposits (arrow). FDG-PET/CT: fluorodeoxyglucose-positron emission tomography/computed tomography

In February 2022, the patient developed symptomatic brain metastasis with meningeal involvement. FDG-PET imaging confirmed extensive metastatic disease involving the liver, lungs, and brain (Figure 2). She was treated with palliative cranial radiotherapy. Subsequent imaging in May 2022 demonstrated further progression of intracranial, pulmonary, and hepatic disease with associated vasogenic oedema, necessitating corticosteroids and antiepileptic therapy.

Following exhaustion of clinical trial options, and given her prolonged disease course and prior treatment-free interval, she was commenced on systemic chemotherapy rechallenge with folinic acid, fluorouracil, and irinotecan (FOLFIRI) in mid-2022. Her disease continued to progress, and she died in October 2022 from complications of metastatic colorectal cancer.

## Discussion

This case represents an uncommon example of prolonged survival in metastatic colorectal cancer with initially unresectable bilobar liver metastases, confirmed at operative exploration. Long-term survival in metastatic colorectal cancer is most frequently documented in patients with limited liver involvement who undergo complete resection, in whom five-year survival rates of approximately 30-40% are routinely observed. Patients presenting with extensive bilobar liver metastases generally have a much poorer prognosis, and survival beyond a decade is rare [1-3,6]. The present case, with survival exceeding 12 years, therefore lies at the extreme upper boundary of reported outcomes for this disease spectrum [1-3,6,7].

Selected series have shown that patients with initially unresectable colorectal liver metastases who respond to systemic therapy and subsequently undergo complete resection can achieve meaningful long-term survival, including survival beyond 10 years in a small subset. These data support aggressive attempts at tumour downstaging and secondary resection in carefully selected individuals [1-3,6,7]. In this context, the current case illustrates how repeated reassessment of resectability - rather than a single, early judgement of unresectability - can create additional opportunities for potentially life-prolonging local treatment [1-3,6-9]. Crucially, the initial assessment of unresectability in this case was not merely a radiologic opinion but was confirmed at operative exploration, lending additional weight to the subsequent achievement of resection following chemotherapy downstaging.

Several factors likely contributed to the prolonged survival observed. First, sustained chemosensitivity across multiple lines of therapy allowed repeated downstaging of the disease and renewed consideration of local treatment over many years. Second, a dynamic surgical strategy encompassing two hepatic resections, complemented by locoregional ablative procedures including RFA and stereotactic body radiotherapy (SBRT) for oligometastatic deposits, provided durable regional control at multiple time points [1-3,6-9]. Third, ongoing multidisciplinary input ensured that systemic therapies, surgery, and local ablative techniques were appropriately sequenced and revisited as the disease and the patient's condition evolved, in line with contemporary guideline-based management principles [2,7-9].

An important contextual observation is that this patient's initial management in 2010 occurred in a regional setting where formalised multidisciplinary tumour board review was not yet standard practice. The initial care pathway was therefore surgeon-led, which was consistent with both the clinical culture and the infrastructure available at that time and location. Despite this, the surgeon appropriately recognised the complexity of the hepatic disease and referred the patient promptly to a tertiary hepatobiliary centre, where the extent of disease was confirmed at operative exploration and resection was correctly abandoned. The patient was subsequently referred to medical oncology for systemic therapy, and care was thereafter coordinated within a multidisciplinary framework. This trajectory underscores the importance of appropriate triage and timely referral even in the absence of a formalised multidisciplinary team (MDT) structure.

It is also important to acknowledge the limitations imposed by the treatment era. The prognostic and predictive significance of primary tumour sidedness in metastatic colorectal cancer - specifically the differential benefit of anti-epidermal growth factor receptor (anti-EGFR) versus anti-vascular endothelial growth factor (anti-VEGF) therapy in left-sided versus right-sided RAS wild-type tumours - was not established until the landmark retrospective analysis of the CALGB/SWOG 80405 trial presented by Venook et al. at the American Society of Clinical Oncology (ASCO) in June 2016 [10,11]. At the time this patient commenced first-line therapy in January 2011, tumour sidedness was not incorporated into biologic agent selection, and bevacizumab-based first-line therapy was the accepted standard of care for RAS wild-type metastatic colorectal cancer. The primary tumour in this case arose in the sigmoid colon, making it a left-sided cancer - a subgroup now recognised to derive particular benefit from anti-EGFR therapy in the first-line setting. This represents an important limitation of the initial treatment selection when viewed in the context of contemporary evidence, though it does not reflect a deviation from the standard of care applicable in 2011.

This case also underscores the importance of individualised decision-making in microsatellite-stable metastatic colorectal cancer, where immunotherapy has historically had limited benefit [2,8-10]. Participation in an early-phase trial of pixatimod (PG545) plus nivolumab highlights the role of innovative strategies in selected patients; in this instance, the patient experienced a meaningful period of disease control before developing immune-related pneumonitis that required treatment discontinuation [7-9,12]. Despite the availability of such trials, referral patterns can be inconsistent, and some clinicians may be reluctant to consider trial enrolment, emphasising the need for greater awareness of appropriate trial options in carefully selected patients. In parallel, the cumulative burden of toxicities from chemotherapy, targeted agents, anticoagulation, and immunotherapy over more than a decade underlines the need to balance potential survival gains against quality of life and to maintain ongoing shared decision-making with patients and their families [2,8-13].

From a broader perspective, this case reinforces that initial unresectability - even when confirmed at operative exploration - does not equate to therapeutic futility in metastatic colorectal cancer. For a minority of patients with favourable tumour biology and sustained treatment responsiveness, repeated downstaging and aggressive local control can achieve survival well beyond what is typically expected [1-3,6-9]. However, these exceptional trajectories should be presented with appropriate caution to avoid creating unrealistic expectations; most patients with extensive bilobar colorectal liver metastases will not experience similar outcomes [1-3,6]. The key message is that careful longitudinal assessment, flexibility in treatment planning, and avoidance of premature therapeutic nihilism are essential to maximising the small but real potential for long-term survival in this challenging clinical context [1-3,6-9].

## Conclusions

This case demonstrates that, in rare and carefully selected patients, metastatic colorectal cancer with initially unresectable bilobar liver metastases - confirmed at operative exploration - can follow a prolonged, relapsing course extending beyond a decade. Sustained chemosensitivity, repeated reassessment of resectability, aggressive local control strategies, and coordinated multidisciplinary care were central to the prolonged survival achieved. The absence of a formalised MDT framework at initial presentation did not preclude the delivery of effective multimodality care, provided appropriate surgical triage and timely oncologic referral were undertaken. Although such outcomes are exceptional and not generalisable, this case emphasises the importance of individualised longitudinal management and avoidance of premature therapeutic nihilism in metastatic colorectal cancer.
